# Cancer statistics in Yemen: incidence and mortality, in 2020

**DOI:** 10.1186/s12889-024-18207-4

**Published:** 2024-04-05

**Authors:** Amran Ibrahim, Marwa El Baldi, Sawsan Mohammed, Karima El Rhazi, Bouchra Benazzouz

**Affiliations:** 1 Department of Epidemiology and Public Health, Faculty of Medicine and Pharmacy, Sidi Mohamed Ben Abdullah University, Fez, Morocco; 2 Department of Biology, Laboratory of Biology and Health, Faculty of Sciences, Ibn Tofail University, Kénitra, Morocco; 3 Department of Hematology, Faculty of Medicine and Health Sciences, Aden University, Aden, Yemen

**Keywords:** GLOBOCAN 2020 database, Cancer statistics, Incidence, Mortality, Yemen

## Abstract

**Background:**

The current cancer epidemiological profile in Yemen suffers from a lack of locally representative data and resources, posing a challenge in determining the real incidence, prevalence, survival and mortality rates, particularly data extracted from national hospitals. This study aims to provide a comprehensive overview of the cancer burden and trends in Yemen for 2020, including incidence and mortality rates.

**Methods:**

The current study provides cancer statistics and their trends in Yemen, including incidence and mortality, in 2020 by using descriptive statistics. The data was obtained using the Global Cancer Observatory (GCO) 2020 online database.

**Results:**

According to the (GCP) database in 2020, the crude incidence rate (CIR) of all cancers in Yemen was 55.2 per 100.000. The age-standardized rate (ASR) was 97.0 per 100.000, and the cumulative risk (0–74) was 22.0 per 100.000. The age-standardized rate (ASR) was 76.5 per 100.000, and the cumulative risk (0–74) was 19.6 per 100.000. Furthermore, the crude mortality rate (CIR) of all cancers was 40.6 per 100.000. Breast cancer was most prevalent in Yemen across all age groups and genders (31.1%), followed by colorectal cancer (7.7%) and leukemia (5.7%). Breast cancer also ranked as the primary cause of mortality at an estimated percentage of 13.5%, followed by colorectal cancer (8.8%) and stomach cancer (7.1%).

**Conclusion:**

Cancer has become a significant life-threatening illness in Yemen with an increase in the disease burden of breast cancer in women. Furthermore, leukemia in children and colorectal cancer in both sexes have experienced a more significant burden as well. Therefore, it is imperative for initiatives for cancer control and prevention to be prioritised at national and regional levels.

## Introduction

Cancer is becoming a big problem in developing countries as life expectancy rises [1]. Cancer surveillance is therefore critical for determining where progress is being achieved and where more needs to be done [2]. The global estimates of cancer are built up from estimates of incidence, prevalence, mortality, and survival in each of the national populations through Population-Based Cancer Registries (PBCR) [3]. Although data from Western countries are regularly included in International Association of Cancer Registries (IARC) publications on cancer incidence, this is not the case for Low- and Middle-Income Countries (LMIC), where the incidence is often estimated, due to the lack of high-quality data from existing Population-Based Cancer Registries (PBCR) [4]. Yemen is a developing country with a variety of meteorological, geographical, and environmental characteristics, and its regions are distinguished by distinct socioeconomic and genetic patterns [5]. Cancer is a significant public health problem in Yemen, and the size of the problem and underlying risk factors is not yet well studied [6]. The true epidemiological profile of cancer in Yemen is unknown due to limited resources for pathology, the scarcity, and quality of medical records, and limited epidemiology resources. On the other hand, war and civil conflict have recently been to the haziness of the national-level cancer burden. In the absence of national cancer surveillance, national cancer registries remain a main challenge in Yemen, as the country lacks a National Cancer Registries Centers [7]. Tobacco smoking is the single most important carcinogenic risk factor in Yemen, contributing to ~ 16.3% of cancers. Overall infections, Hepatitis B infection and obesity, and occupational risks is another important preventable cancer contributor which are responsible for ~ 13.4, 2.5% and, 1.4% respectively. Effective tobacco control policies, suggestions for healthier lifestyles, as well as expanding the coverage of successful screening, educating, and immunization programs, should all be included in comprehensive prevention and control strategies in Yemen in order to better sensitize greater awareness control to the general public. The updating of national cancer profiles and shedding more light on cancer risk factors would provide basic know hows on how to tackle it more effectively and can give scientific data for Yemen’s health policymaking [8–10].

## Materials and methods

Data sources were extracted from the GLOBOCAN 2020 online database Cancer Today (https://gco.iarc.fr/today/home). GLOBOCAN estimates of the incidence, mortality, and prevalence of 36 cancer types in 185 countries or territories, categorized by sex and age group.

IARC’s approach is to evaluate, compile and use data provided by IARC collaborators in all countries of the world and also includes working alongside national staff to improve local data quality, record coverage and analytical capacity.

The estimation methods are specific to each country, and the quality of national estimates depends on the coverage, accuracy, and timeliness of recorded incidence and mortality data. The aim is to inform cancer control by improving the coverage, quality, and use of population-based cancer [11–13].

### Statistical analysis

The incidence and mortality rates observed most recently at the national level were applied to the 2020 population. Rates were estimated by modelling national mortality data, using mortality-to-incidence ratios derived from the national cancer registries. National incidence and mortality rates for all cancers combined were obtained by averaging age- and sex-specific rates. These rates were then used to determine the incidence of specific cancer sites based on available cancer-specific relative frequency data. An age-standardized rate (ASR) is a weighted average of the age-specific rates, with the weights being based on the population distribution of a standard population. The age-standardized incidence or mortality rate (W) is the calculated rate expressed per 100.000person-years12. The crude incidence rate is calculated by dividing the total number of new cancer cases diagnosed in a specific year in the population category of interest by the at-risk population for that category, multiplying the result by 100.000. This rate is a useful measure for comparing cancer incidence across different populations. It is important to note that this rate does not take into account differences in age, sex, or other factors that may affect cancer incidence. A crude death rate equals the total number of cancer deaths during a specific year in the population category of interest, divided by the at-risk population for that category and multiplied by 100.000. Descriptive analysis was conducted on the extracted data, followed by comparisons and discussions. Due to the descriptive nature of the study, it was not possible to provide an accurate profile for the incidence and mortality of cancers in Yemen [11–13].

## Results

### Cancer incidence in Yemen during 2020

**Fig. 1:**
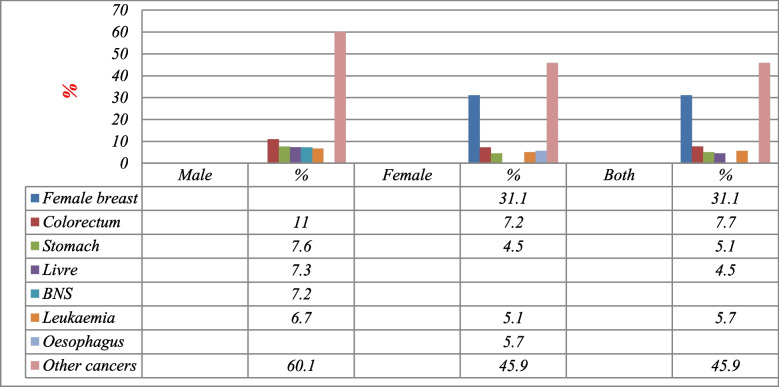
Percentage of cancer incidence in all ages (both genders, males and females), Yemen, 2020. Data sources: https://gco.iarc.fr/today/home

According to GLOBOCAN 2020 data statistics, the crude incidence rate (CIR) of all cancers was 55.2/100.000. The age-standardized rate (ASR) was 97.0/100.000, and the cumulative risk (0–74) was 22.0/100.000, (Table 1). Overall, it was registered 16,476 new cases distributed as follows: female breast (31.1%), colorectum (7.7%), leukaemia (5.7%), stomach (5.1%), non-Hodgkin lymphoma (4.5%), and other cancers (45.9%). Besides, breast cancer was ranked first in Yemen with ASR 30.5/100.000 population, followed by colorectum (10.7/100.000), leukaemia (4.2/100.000), stomach (7.1/100.000), Non-Hodgkin Lymphoma (4.0/100.000), oesophagus (6.4/100.000), BNS (3.8/100.000), lung (5.8/100.000), liver (5.1/100.000), Thyroid (1.9/100.000), hodgkin lymphoma (2.7/100.000), nasopharynx (1.7/100.000), pancreas (2.0/100.000), larynx (2.5/100.000), and ovary (2.2/100.000population) (Table 2 and Fig. 1).

**Table 1:** Summary of cancer statistics for all ages in Yemen, 2020, GLOBOCAN estimates

Incidence	Item	Both sexes	Males	Females
	Number of new cases	16,476	7159	9317
	Crude rate	55.2	47.6	62.9
	ASR (World) per 100,000	97.0	92.7	102.2
	Cumulative risk (0–74),	22.0	23.2	21.3
Mortality	Number of deaths	12,103	5667	6436
	Crude rate	40.6	37.7	43.5
	ASR (World) per 100,000	76.5	77.9	76.1
	Cumulative risk (0–74)	19.6	21.3	18.5
Prevalence (5-year)	Number of prevalent cases	26,651	11,158	15,493
	Proportion	89.4	74.3	104.7
Risk of developing cancer before the age of 75 years %		9.8	9.3	10.3
Risk of dying from cancer before the age of 75 years %		7.9	7.9	7.9

Data sources: https://gco.iarc.fr/today/home

**Table 2:** Number of incidences, percentage, CIR, ASR, and Cumulative Rates (per 100,000) among both genders for all ages by 10 primary cancer sites, Yemen, 2020

Site	Male					Site	Femal					Site	Both genders				
	*N*	%	Crude rate	ASR (World) per 100,000	Cumulative risk (0–74)		*N*	%	Crude rate	ASR (World) per 100,000	Cumulative risk (0–74)		*N*	%	Crude rate	ASR (World) per 100,000	Cumulative risk (0–74)
Colorectum	790	11.0	5.3	12.0	–	Breast	2894	31.1	19.6	30.5	6.0	Breast	2894	17.6	19.5	30.5	6.0
Stomach	546	7.6	3.6	8.9	3.8	Colorectum	722	7.7	4.9	9.5	–	Colorectum	1512	9.2	5.1	10.7	–
NHL	526	7.3	3.5	5.0	0.93	Oesophagus	532	5.7	3.6	7.2	2.6	Leukaemia	988	6.0	3.3	4.2	0.66
Leukaemia	514	7.2	3.4	4.4	0.67	Leukaemia	474	5.1	3.2	4.0	0.65	Stomach	966	5.9	3.2	7.1	2.7
BNS	478	6.7	3.2	4.9	1.4	Stomach	420	4.5	2.8	5.7	1.9	NHL	862	5.2	2.9	4.0	0.63
Liver	459	6.4	3.1	7.1	2.2	Thyroid	418	4.5	2.8	3.9	0.44	Oesophagus	859	5.2	2.9	6.4	2.3
Lung	425	5.9	2.8	6.7	2.8	Lung	359	3.9	2.4	5.1	2.0	BNS	803	4.9	2.7	3.8	0.83
Oesophagus	327	4.6	2.2	5.3	1.9	NHL	336	3.6	2.3	3.2	0.40	Lung	784	4.8	2.6	5.8	2.3
Larynx	313	4.4	2.1	4.4	0.91	Ovary	329	3.5	2.2	3.4	0.46	Liver	745	4.5	2.5	5.1	1.5
Nasopharynx	285	4.0	1.9	3.0	0.34	BNS	325	3.5	2.2	2.9	0.42	Thyroid	560	3.4	1.9	2.7	0.32
Other cancers	2496	34.8	–	–	–	Other cancers	2508	26.9	–	–	–	Other cancers	5503	33.5	–	–	–

Data sources https://gco.iarc.fr/today/home

In males, there were a total of 7159 new cases distributed as follows: colorectum (11.0%), stomach (7.6%), non-Hodgkin lymphoma (7.3%), leukaemia (7.2%), brain and central nervous system (6.7%), and other cancers (60.1%). In this group, the CIR of all cancers was 47.6/100.000, the ASR was 92.7/100.000, and the cumulative risk (0–74) was 23.2/100.000, (Table 2 and Fig. 1). Among the Yemeni population of males, colorectum cancer was ranked first in Yemen with ASR 12.0/100.000 population, followed by stomach (8.9/100.000), NHL (5.0/100.000), leukaemia (4.4/100.000), BNS (4.9/100.000), liver (7.1/100.000), lung (6.7/100.000), oesophagus (5.3/100.000), larynx (4.4/100.000), nasopharynx (3.0/100.000), hodgkin lymphoma (2.1/100.000), bladder (4.0/100.000), prostate (2.8/100.000), pancreas (2.5/100.000), and thyroid (1.5/100.000population) (Table 2 and Fig. 1).

In females, there were a total of 9317 new cases distributed as follows: breast cancer (31.1%), colorectum (7.7%), oesophagus (5.7%), leukaemia (5.1%), stomach (4.5%) and other cancers (45.9%). In this group, the CIR of all cancers was 62.9/100.000; the ASR was 102.2/100.000; and the cumulative risk (0–74) was 21.3/100.000. Among the Yemeni population of females, breast cancer was ranked first in Yemen with ASR 30.5/100.000population, followed by colorectum (9.5/100.000), oesophagus (7.2/100.000), leukaemia (4.0/100.000), stomach (5.7/100.000), thyroid (3.9/100.000), lung (5.1/100.000), NHL (3.2/100.000), ovary (3.4/100.000), BNS (2.9/100.000), liver (3.5/100.000), cervix uteri (2.5/100.000), pancreas (2.4/100.000), lip, oral cavity (1.8/100.000), and hodgkin lymphoma (1.2/100.000 population), (Table 2 and Fig. 1).

Tables 2 also show the common of the 15 cancers in both male and female genders, separately, the number of incidences, percentage, CIR, ASR, and cumulative rates (per 100.000) for all ages.

### Cancer mortality in Yemen during 2020

**Fig. 2:**
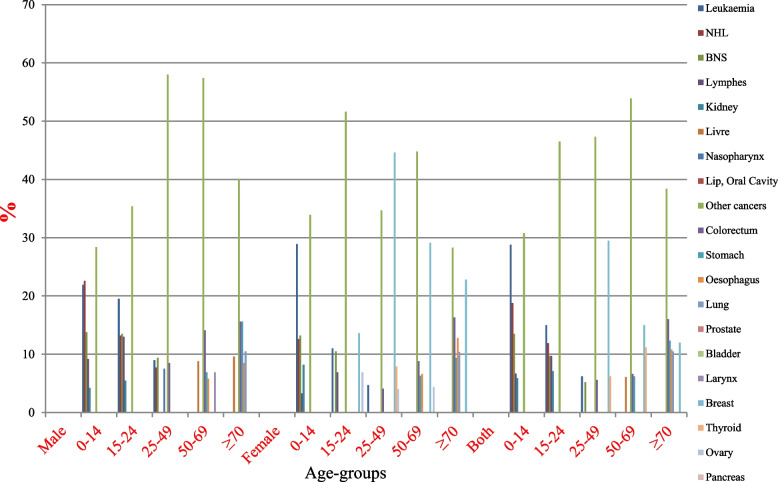
The 5 common types of cancer death in males, female and both. Data sources: https://gco.iarc.fr/today/home

**Fig. 3:**
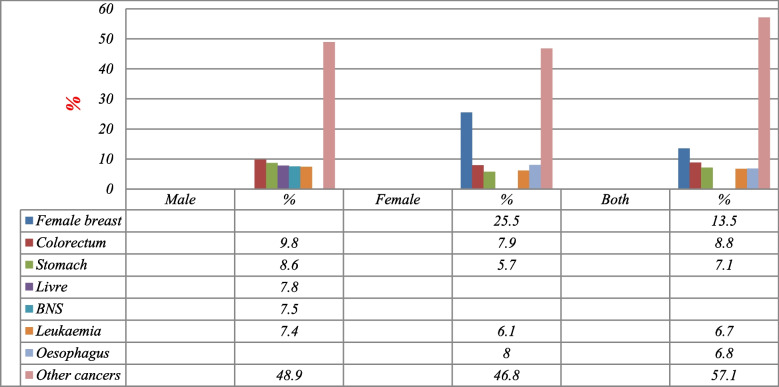
Percentage of cancer mortality in all ages (both genders, males and females), Yemen, 2020. Data sources: https://gco.iarc.fr/today/home

According to GLOBOCAN 2020 data statistics, the CIR of all cancers was 40.6/100.000; the ASR was 76.5/100.000, and the cumulative risk (0–74) was 19.6/100.000, (Table 1 and Fig. 2). Overall, it was registered 12,103 new deaths distributed as follows: breast cancer (13.5%), colorectum (8.8%), stomach (7.1%), oesophagus (6.8%), leukaemia (6.7%), and other cancers (57.1%). Furthermore, breast cancer was ranked first in Yemen with ASR 18.9/100.000 population, followed by colorectum (7.7/100.000), stomach (6.4/100.000), oesophagus (6.2/100.000), leukaemia (3.6/100.000), lung (5.5/100.000), liver (5.0/100.000), BNS (3.5/100.000), NHL (3.0/100.000), nasopharynx, (1.7/100.000) pancreas (2.4/100.000), larynx (1.8/100.000), ovary (2.9/100.000), bladder (1.6/100.000), and lip, oral cavity (1.3/100.000 population) (Table 3 and Fig. 3).

**Table 3:** Number of deaths, percentage, CIR, ASR, and Cumulative Rates (per 100,000) among both genders for all ages by 10 primary cancer sites, Yemen, 2020

Site	Male					Site	Female					Site	Both Genders				
	*N*	%	Crude rate	ASR (World) per 100,000	Cumulative risk (0–74)		*N*	%	Crude rate	ASR (World) per 100,000	Cumulative risk (0–74)		*N*	%	Crude rate	ASR (World) per 100,000	Cumulative risk (0–74)
Colorectum	554	9.8	3.7	8.6	–	Breast	1638	25.5	11.1	18.9	4.7	Breast	1638	13.5	11.1	18.9	4.7
Stomach	488	8.6	3.2	8.0	3.4	Oesophagus	516	8.0	3.5	7.0	2.6	Colorectum	1063	8.8	3.6	7.7	–
Liver	442	7.8	2.9	7.0	2.2	Colorectum	509	7.9	3.4	6.9	–	Stomach	858	7.1	2.9	6.4	2.4
BNS	425	7.5	2.8	4.7	1.4	Leukaemia	390	6.1	2.6	3.4	0.60	Oesophagus	828	6.8	2.8	6.2	2.3
Leukaemia	419	7.4	2.8	3.8	0.65	Stomach	370	5.7	2.5	5.1	1.7	Leukaemia	809	6.7	2.7	3.6	0.62
Lung	405	7.1	2.7	6.5	2.7	Lung	329	5.1	2.2	4.8	1.8	Lung	734	6.1	2.5	5.5	2.2
NHL	372	6.6	2.5	3.9	0.83	Liver	271	4.2	1.8	3.4	0.88	Liver	713	5.9	2.4	5.0	1.4
Oesophagus	312	5.5	2.1	5.2	1.8	BNS	264	4.1	1.8	2.5	0.40	BNS	689	5.7	2.3	3.5	0.83
Larynx	261	4.6	1.7	3.8	0.85	Ovary	244	3.8	1.6	2.9	0.44	NHL	591	4.9	2.0	3.0	0.53
Nasopharynx	233	4.1	1.5	2.6	0.30	NHL	219	3.4	1.5	2.2	0.32	Nasopharynx	326	2.7	1.1	1.7	0.19
Other cancers	1756	30.6	–	–	–	Other cancers	1686	25.4	–	–	–	Other cancers	3854	31.9	–	–	–

Data sources: https://gco.iarc.fr/today/home

In males, there were a total of 5667 deaths distributed as follows: colorectum (9.8%), stomach (8.6%), liver (7.8%), BNS (7.5%), leukaemia (7.4%), and other cancers (58.9%). In this group, the CIR of all cancers was 37.7/100.000, the ASR was 77.9/100.000 and the cumulative risk (0–74) was 21.3/100.000. Among the Yemeni population of males, colorectum cancer was ranked first in Yemen with ASR 8.6/100.000 population, followed by stomach (8.0/100.000), liver (7.0/100.000), BNS (4.7/100.000), leukaemia (3.8/100.000), lung (6.5/100.000), NHL (3.9/100.000), oesophagus (5.2/100.000), larynx (3.8/100.000), nasopharynx (2.6/100.000), bladder (2.9/100.000), pancreas (2.4/100.000), hodgkin hymphoma (1.3/100.000), prostate (1.7/100.000), and lip, oral cavity (1.1/100.000 population) (Table 3 and Fig. 3).

In females, there were a total of 6436 new deaths distributed as follows: breast (25.5%), oesophagus 516 (8%), colorectum (7.9%), leukaemia (6.1%), 370 (5.7%), other cancers (46.8%). In this group, the crude mortality rates (CIR) of all cancers was 43.5/100.000, the ASR was 76.1/100.000, and the cumulative risk (0–74) was 18.5/100.000. among the Yemeni population of females, breast cancer was ranked first in Yemen with ASR 18.9/100.000 population, followed by colorectum (7.0/100.000), oesophagus (6.9/100.000), leukaemia (3.4/100.000), stomach (5.1/100.000), lung (4.8/100.000), liver (3.4/100.000), BNS (2.5/100.000), ovary (2.9/100.000), NHL (2.2/100.000), pancreas (2.4/100.000), cervix uteri (1.8/100.000), lip, oral cavity (1.4/100.000), thyroid (1.3/100.000) and nasopharynx (0.87/100.000population), (Table 3 and Fig. 3).

### Age specific in cancer incidence and mortality

#### ASR (age-standardised rate); incidence

Categories were created for 5 age intervals spanning childhood and adulthood: ages < 15 years, ages 15–24 years, ages 25–49 years, ages 50–69 years, ages ≥70 years. As a result, each age-group cohort demonstrated some characteristics in the most frequent type of cancer, with similarities and variances between males and females.

**Fig. 4:**
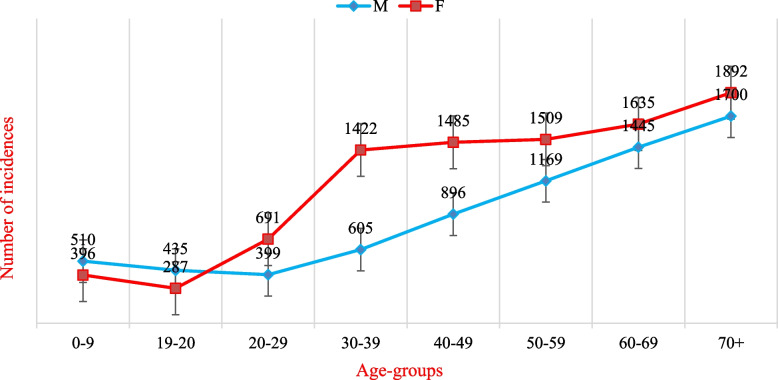
The 5 common types of cancer incidence in males, female and both. Data sources: https://gco.iarc.fr/today/home

Overall, leukaemia was the most common type of cancer in children and teenagers aged < 25 years with a rate of (24.8–15.0%) respectively and female breast cancer was the most incidence common in the two cohorts aged (25–49) and (50–69) years with a rate of (29.9–15.9%). In the cohort belonging to the elderly cohort aged ≥70 years colon cancer was the most common type of cancer with a rate of (16.0%) (Fig. 4).

In males, NHL and leukaemia were the most common type of cancer in children aged < 15 years with a rate of (22.6–21.9% respectively) and leukaemia cancer also was the most incidence common in the teenagers 15–24 years of age with a rate of (19.9%). While BNS was the most incidence common in the cohort belonging to the 25–49 year group. While colorectal cancer was the most incidences common type of cancer in the cohorts aged (50–69) years with a rate of (14.1%). In the cohort belonging to the elderly cohort aged ≥70 years stomach and colorectal cancer was the most incidence common with a rate of (15.6% for both) (Fig. 4).

In females, leukaemia was the most common type of cancer in children aged < 15 years with a rate of (28.9%) and breast cancer was the most common type of cancer in the other three age-cohorts (15–24, 25–49, and 50–69) with a rate of (13.6%), 44.6 and 29.1% respectively). While in the cohort belonging to the elderly cohort aged ≥70 years breast also and colorectal cancer were the most common type of cancer with a rate of (22.8 and 16.3% respectively) (Fig. 4).

**Fig. 5:**
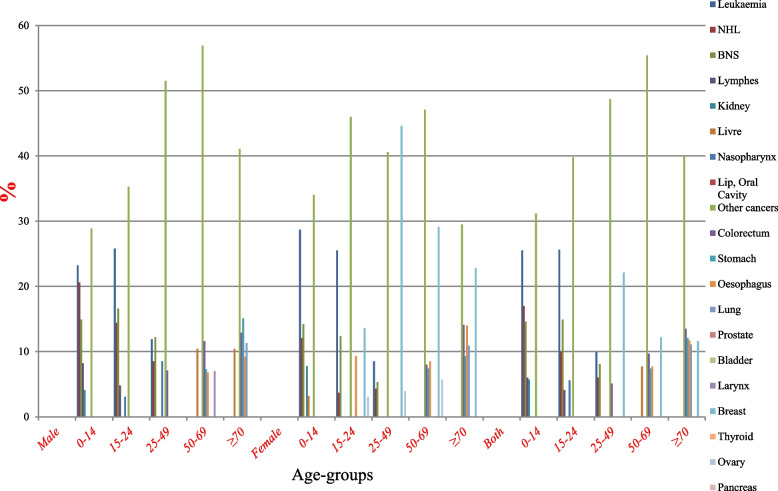
The number of cancer cases with age among Yemeni males and females. Data sources: https://gco.iarc.fr/today/home

Figure 5 indicates that the incidence of cancer grows with age in both sexes, with males having a greater incidence rate than females in the childhood category. But was then surpassed by higher incidences in females, especially in those age groups (30--50 years). This can be explained by the high incidence of breast cancer among females, and then the incidence rates for both sexes continue to grow in those over 60 years of age and over for both sexes, with light raise in incidence rates among females.

#### ASR (age-standardised rate); mortality

Five taxonomic categories were used to gain better knowledge of the most common cancers deaths by sex: children < 15 years of age; adolescents and adolescents between the ages of 15 and 24; young people from 25 to 49 years old; adults 50–69 years old; and the elderly ≥70 years old. As a result, each age-group cohort demonstrated some characteristics in the most frequent type of cancer, with similarities and variances between males and females.

Overall, leukaemia was the deadliest common type of cancer in children and teenagers aged < 25 years with a rate of (25.6–25.5%) respectively and female breast cancer was the deadliest common type of cancer in the two cohorts aged (25–49 and 50–69) years with a rate of (22.1–22.2%), While in the cohort belonging to the elderly cohort aged ≥70 years colorectal cancer was the deadliest common with a rate of (13.5%) (Fig. 2).

In males, NHL and leukaemia were the deadliest common type of cancer in children aged < 15 years with a rate of (23.2–20.6%, respectively) and leukaemia cancer also was the deadliest common in teenagers (15–24) years of age with a rate of (25.8%). While BNS and leukaemia cancer were the most death common in the cohort belonging to the (25–49) year group with a rate of (12.2 and 11.9%, respectively) and colorectal and liver cancer were the deadliest common type of cancer in the cohorts aged (50–69) years with a rate of (11.6 and 10.4%, respectively). In the cohort belonging to the elderly cohort aged ≥70 years stomach and colorectal cancer was the deadliest common with a rate of (15.1 and 12.9% for, respectively) (Fig. 2).

In females, Leukaemia was the deadliest common type of cancer in children and teenagers aged < 25 years with a rate of (28.7% and 25.5, respectively). While breast cancer was the deadliest common type of cancer in the two age-cohorts (25–49 and 50–69) with a rate of ((37.4%) and (23.2%)), respectively). While in the cohort belonging to the elderly cohort aged ≥70 years breast also, colorectal, and oesophagus cancer were the deadliest common type of cancer with a rate of (22.2, 14.1, and 14.0% respectively) (Fig. 2).

**Fig. 6:**
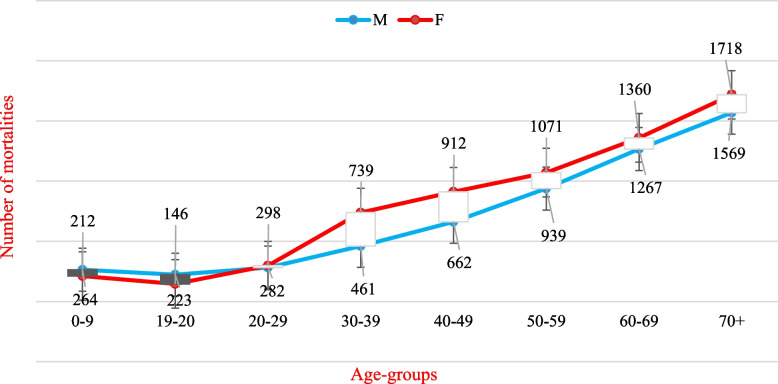
The number of cancer deaths with age among Yemeni males and females. Data sources: https://gco.iarc.fr/today/home

Figure 6 indicates that the death of cancer grows with age in both sexes, with males having a little greater death rate than females in the childhood category. But was then surpassed by higher deaths in females, especially in those age groups (30--60 years). This can be explained by the high death of breast cancer among females, and then the death rates for both sexes continue to grow in those over 60 years of age and over for both sexes, with light raise in death rates among females.

Tables 3 also show the common of the 10 cancers in both male and female genders, separately, the number of deaths, percentage, CIR, ASR, and Cumulative Rates (per 100.000) for all ages.

## Discussion

Annual cancer statistics surveillance is critical for determining where progress is being achieved and where more needs to be done [2]. Cancer-control initiatives are having an effect on Yemen’s cancer burden. However, cancer remains a growing burden on the Yemeni healthcare system, with an annual increase in the total number of new primary cancers expected, owing in large part to population growth and age [7]. In this paper, we reported the overview of cancer burden and their trends in Yemen, including incidence and mortality, in 2020 using GLOBOCAN 2020 online data source. There were 16,476 cases and 12,103 deaths registered among both genders of all ages.

Overall, cancer cases were more among females than males. Comparisons showed that cases of breast cancer were dominant among females in Oman, Saudi Arabia, Egypt, Morocco, Britain, and America, with estimates of (37.7%), (29%), (32.4%) (38.9%), (25.5%), and (24%), respectively by the GLOBOCAN 2020 database. While there were some differences in dominant cases among males, colorectal was dominant in Oman and Saudi Arabia, (12.9%), (19.3%) respectively, liver (27.3%) in Egypt, lung (22.3%) in Morocco, prostate in Britain and America; (23.1%), (17.1%), respectively [1, 5, 6, 14].

Contrary to data indicating that lung and colorectal cancer are the most frequent types of cancer in high-income nations and the Gulf of Bahrain, it is surprising that the incidence of lung cancer is not highly reported in the GLOBOCAN 2020 database. This might be the result of Yemenis having a reduced life expectancy and poor lung cancer diagnosis, with a focus on tuberculosis rather than lung cancer [3, 11, 15]. But according to the most recent world health organization (WHO) report on the global tobacco epidemic, published in 2017, Yemen’s prevalence rate of tobacco use has climbed by 18.7%, contributing to ~ 16.3% of cancers. Smoking in the nation is frequently connected to locally utilized leaves called Khat (*Catha edulis*), which may cause lung cancer rates to rise in the future [16, 17].

**Fig. 7:**
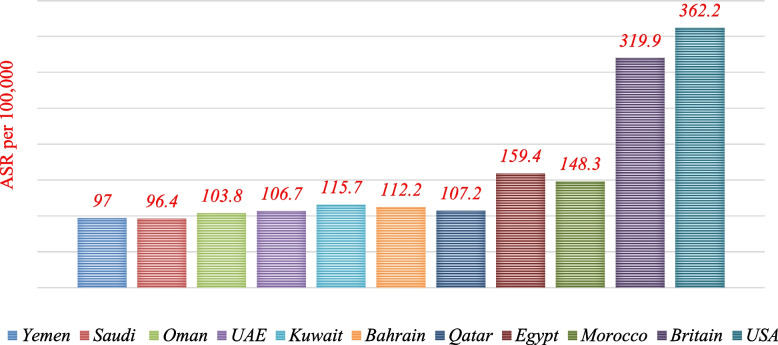
Comparing the ASR (World) per 100,000 for incidence, both sexes, and all ages in Yemen with some international rates. Data sources: https://gco.iarc.fr/today/home

The range of ASR among the 15 most common cancer sites in males ranged from (12.0/100.000 population) for colorectum cancer to (1.5/100.000 population) for thyroid cancer, while in females it was between (30.5/100.000 population) for breast cancer and (1.2/100.000 population) for hodgkin lymphoma cancer. Although female breast cancer was the most common cancer in Yemen, the rate was the lowest compared with the selected world countries. Moreover, the ASR of breast cancer in Yemen was three times lower than in the United States of America cancer (90.3/100.000 vs. 30.5/100.000). Similarly, colorectal cancer in males was three times lower than in the United Kingdom of Britain (34.1/100.000 vs. 10.7/100.000) [18]. The population in the chosen countries with higher rates of cancer incidence probably goes through some kind of screening and early detection for cancer, which is missing in Yemen. Furthermore, primary healthcare services in Yemeni ministry of health areas are not comparable to those in selected countries where basic screening methods for breast cancer, prostate cancer, and colorectal cancer are practiced on a daily basis [19]. Comparisons showed that the ASR incidence rate of different types of cancers (per 100.000 population) in Yemen is less or equal to data in some selected countries in the Middle East and others (Fig. 7). This is of course due to the fact that the reported cases in Yemen do not correspond to an actual number of cases. In addition to the misdiagnosis of some cancerous tumors, the discovery of cancer in very late stages, the scarcity of epidemiological and statistical studies, limited resources for pathology, and the scarcity of medical registries. On the hand, some national studies showed lower rates than GLOBOCAN estimates for 2020 [1, 5, 14, 18].

With comparing the proportion rates of similar types of cancers between females and males except for breast cancer, which is sex-specific and dominant in females, in the age group (0–14) females showed higher rates of leukemia cancers compared with males (28.9% females) vs. (21.9% males), while in age groups (15–24 and 25–49) males showed higher rates of leukemia and BNS compared with females (19.5 and 13.5% males) vs. 11 and 10.5% females) respectively, and also in the age group 50–69 males showed higher rates of colorectal compared with females (14.1% males) vs. (8.8% females) (Fig. 4). Surprisingly, through the distribution of cancers in the children’s age groups in both genders, childhood cancer is leukemia, which corresponds to the most prevalent cancers in Yemeni childhood reported in prior studies, with leukaemia being the most common, followed by lymphoma and central nervous system tumors (Fig. 2) [3, 6, 20]. Except for breast cancer in females, which is the most frequent in many world countries, data from other Middle Eastern countries, such as Saudi Arabia, Jordan, Morocco, and Egypt, show patterns of cancer incidence that differ from data from Yemen [21–25].

According to the age groups in both sexes, Leukaemia was the most common type of cancer in children and teenagers aged < 25 years with a rate of (24.8–15.0%) respectively and female breast cancer was the most incidence common in the two cohorts aged (25–49) and (50–69) years with a rate of (29.9–15.9%). In the cohort belonging to the elderly cohort aged ≥70 years colon cancer was the most common type of cancer with a rate of (16.0%). The data not only emphasize the population’s cancer burden as a health priority, but also emphasize the importance of understanding variations over time and that there may be minor differences in rankings between areas within the same country and city as a result of lifestyle, population quality of life and behavioral risk factors, and different environmental exposures.

Figure 5 indicates that the incidence of cancer grows with age in both sexes, with males having a greater incidence rate than females in the childhood category. But was then surpassed by higher incidences in females, especially in those age groups (30--50 years). This can be explained by the high incidence of breast cancer among females, and then the incidence rates for both sexes continue to grow in those over 60 years of age and over for both sexes, with light raise in incidence rates among females. According to El-Zaemey (2012) of the National Oncology Centre in Sana’a, Yemen, roughly 71% of women diagnosed with breast cancer were 50 or younger. In the same context [26], other studies have addressed the involvement of reproductive factors in the development of breast cancer, such as prolonged oestrogen exposure, early menarche, late menopause, and others [19, 27, 28]; however, these factors were not studied in Yemen.

It is difficult to determine the specific mortality rates for cancer, especially for those coming from remote areas of Yemen. Thus, it becomes difficult for them to access the healthcare centers in the country’s main cities [11, 29].

Overall, cancer deaths were more among females than males. Comparisons showed that death from breast cancer was dominant among females in Oman, Saudi Arabia, and Morocco, with (37.7%), (29%), and (24.7%), respectively, while liver (22.9%) in Egypt, lung in Britain and America (20.8%), (22.5%), respectively by data 2020. While among males, colorectal was dominant in Oman and Saudi Arabia, (11.9%) and (15.8%) respectively, liver (35.6%) in Egypt, lung in Morocco, Britain, and America (28.5%), (22.7%), and (22.7%), respectively [11].

**Fig. 8:**
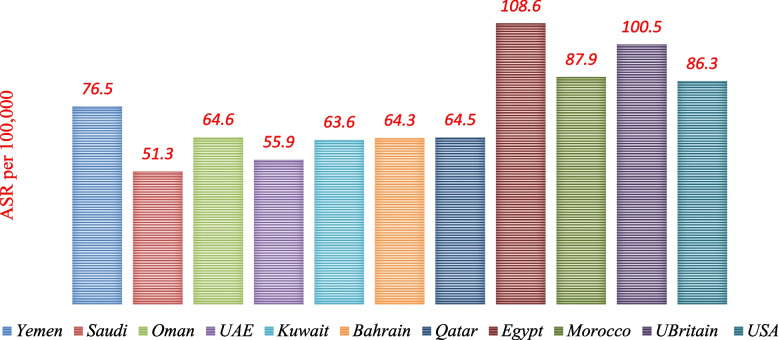
Comparing the ASR (World) per 100,000 for mortality, both sexes, and all ages in Yemen with some international rates. Data sources: https://gco.iarc.fr/today/home

Comparisons showed that the ASR mortality rate of different types of cancers (per 100.000populations) in Yemen is less or equal to data in some selected countries in the Middle East and others (Fig. 8). It is difficult to determine the precise mortality rates for cancer, especially for those coming from remote regions of Yemen. That is due to the fact that the reported cases in Yemen do not correspond to an actual number of cases. In addition to the misdiagnosis of some diseases as the fact of death and the “underlying” cause of death is not established, the discovery of cancer in very late stages, the scarcity of epidemiological and statistical studies, limited resources for pathology, and the scarcity of medical registries. On the hand, some national studies showed lower rates than GLOBOCAN estimates for 2020.

With comparing the deadliest common type of cancer between females and males except for breast cancer, which is sex-specific and dominant in females in the cohort belonging to (25*–*49 and 50*–*69), leukaemia was the deadliest common type of cancer in the cohort belonging to (0*–*24) with a rate of (28.7% females) vs. (23.2% males), while in the cohort belonging to the elderly cohort aged ≥70 years colorectal cancer was the deadliest common with a ratio of (22.2%*)* among females compared with stomach cancer with a ratio of (15.1%) among males (Fig. 2) [3, 20]*.* However, no significant differences in cancer death were seen between males and females. Data from other Middle Eastern countries, such as Saudi Arabia, Jordan, Morocco, and Egypt, show patterns of cancer death that agree with the data from Yemen [21, 22, 24, 30, 31].

It has been clearly established that developing countries, such as Yemen, have low accuracy in determining the underlying cause of death, compared to high accuracy in developed countries. Furthermore, many nations in the Eastern Mediterranean Region and North Africa lacked the financial and technological resources needed to collect high-quality, complete, and timely data on mortality [32–35]. However, using death certificates and estimating cancer-related death rates may be a crucial step in extrapolating Yemen’s cancer mortality in order to activate the existing comprehensive national cancer control program, which includes an active role in cancer surveillance, and integrate it into the country’s existing healthcare system. Unfortunately, full data on cancer incidence and death for the entire country are not yet accessible because the Ministry of Health in Yemen’s registries are only partially operational due to a lack of technical and financial support. However, estimating Yemen’s cancer profile might be conceivable if GLOBOCAN 2020 data were utilized to extrapolate Yemen’s cancer mortality and incidence.

It is important to note that: Five population-based cancer registries were established in Yemen to collect, operate, and evaluate data on cancer patients. These include the Aden Cancer Registry (ACR), overseen by Aden University and accepted by the International Association of Cancer Registries in 1998, and the National Oncology Center in Sana’a, which is administratively affiliated with the National Cancer Foundation (NCCF). The Yemen Cancer Registry (YCR) was established in 2003 and has branches in several provinces. Additionally, there are three other registries: the Hadhramaut Cancer Registry in Al-Mukalla City, the Hadramout Valley and Desert Oncology Center (HVDOC), and the Taiz Cancer Registry (administratively affiliated with the Ministry of Public Health and Population). However, the final three registries only serve as hospital registries.

It is also important to mention the cancer risk factors in Yemen in this aryical. Limited research has been conducted on cancer risk factors in Yemen. Only one study has explored breast cancer risk factors, which identified several primary risk factors among Yemeni women. These include being divorced, never having breastfed a child, using oral contraceptives, hypertension, having a family history of malignancy, and postmenopausal status. Another study used data mining techniques to investigate cancer risk factors. The study results indicate that smoking, chewing tobacco (Shamaa), age, marital status, and place of residence are the most significant risk factors for developing cancer. Another study focused on gastric cancer risk factors found that living in rural areas, tobacco chewing (Shamma), and drinking untreated water are the main contributors to this disease. Various studies have been conducted on public awareness and knowledge of cancer risk factors, including breast, oral, lung and other cancers. References suggest that in Yemen, the primary risk factors for cancer are tobacco use (16.3%), overall infections (13.4%), Hepatitis B infection, obesity (2.5%), and occupational hazards (1.4%) [9, 10, 36].

## Conclusions

Population growth and aging, together with lower infectious disease mortality, lifestyle, and different environmental exposures, could explain why cancer incidence has grown globally. Cancer is becoming a big problem in developing countries mainly due to life expectancy rises, and a growing, and aging population too [7, 37, 38]. The cancer registries in LMIC, often struggle with lack of funding and incomplete or inaccurate data. In addition, many countries in Eastern Mediterranean Region and North Africa were lacked financial or technical resources to collect good quality, complete and timely data [32–34]. The exact cancer incidence and mortality in Yemen is unknown, often hidden and diagnosed in late stages due to limited resources for pathology, the scarcity and quality of medical records, absence of early diagnostic devices, and limited epidemiology resources. There aren’t enough Yemeni national statistics on cancer, and information on cancer patterns is still very limited [6, 39, 40].

Estimates show areas where more work is required. Currently, no screening tests are available for primary cancers except female breast cancer. Greater efforts are required to improve primary prevention, early detection, and treatment for major cancers, particularly with the introduction of technical advancements in the management of these advanced tumors since the 1990s, as alternatives for detection and treatment remain so limited. To further lessen the effects of cancer on individuals in Yemen, more support and funding must be provided for creative research projects and healthy, effective public policies that are implemented across the spectrum of cancer prevention.

There are a few limitations to this study. Due to the descriptive character of the study; this could not give real profile for further rate of cancer in the Yemen. The IARC GLOBOCAN-Yemen-2020 estimates for incidence and mortality of cancer were derived from data available from the national cancer registries.

Estimates indicate that cancer has a substantial effect on the Yemeni population. There is a critical imperative for enhanced public education and training to bridge the expanding knowledge gap between evidence-based cancer prevention and the reduction of established risk factors. It is crucial to establish effective policies for controlling known risk factors and to prioritise healthy lifestyles. Moreover, there is a need to broaden the reach of successful screening, awareness-raising, and vaccination programmes. Ultimately, prevention is preferable to treatment.

## Data Availability

All data generated or analyzed during this study are available in the GLOBCAN 2020 online database (gco.iarc.fr) or in the referenced published articles.
